# Structures of the essential efflux pump EfpA from *Mycobacterium tuberculosis* reveal the mechanisms of substrate transport and small-molecule inhibition

**DOI:** 10.21203/rs.3.rs-3740027/v1

**Published:** 2024-01-05

**Authors:** Shuhui Wang, Kun Wang, Kangkang Song, Pengfei Li, Dongying Li, Yajie Sun, Ye Mei, Chen Xu, Maofu Liao

**Affiliations:** 1 Department of Cell Biology, Harvard Medical School, Boston, MA, USA.; 2 Department of Molecular Metabolism, Harvard T.H. Chan School of Public Health, Boston, MA, USA.; 3 Department of Biochemistry & Molecular Biotechnology, University of Massachusetts Chan Medical School, Worcester, MA, USA; 4 Cryo-EM Core Facility, University of Massachusetts Medical School, Worcester, MA, USA.; 5 Single Particle, LLC, San Diego, CA, USA.; 6 Cancer Center, Union Hospital, Tongji Medical College, Huazhong University of Science and Technology, Wuhan, China.; 7 State Key Laboratory of Precision Spectroscopy, School of Physics and Electronic Science, East China Normal University, Shanghai, China.; 8 Present address: Cryo-electron microscopy center, Southern University of Science and Technology, Shenzhen, China.; 9 Present address: Department of Molecular Biophysics and Biochemistry, Yale University, New Haven, USA.; 10 Present address: Institute for Biological Electron Microscopy, Southern University of Science and Technology, Shenzhen, China.; 11 Present address: Department of Chemical Biology, School of Life Sciences, Southern University of Science and Technology, Shenzhen, China.

## Abstract

As the first identified multidrug efflux pump in *Mycobacterium tuberculosis* (*Mtb*), EfpA is an essential protein and promising drug target. However, the functional and inhibitory mechanisms of EfpA are poorly understood. Herein we report cryo-EM structures of EfpA in outward-open conformation, either bound to three endogenous lipids or the inhibitor BRD-8000.3. Three lipids inside EfpA span from the inner leaflet to the outer leaflet of the membrane. BRD-8000.3 occupies one lipid site at the level of inner membrane leaflet, competitively inhibiting lipid binding. EfpA resembles the related lysophospholipid transporter MFSD2A in both overall structure and lipid binding sites, and may function as a lipid flippase. Combining AlphaFold-predicted EfpA structure, which is inward-open, we propose a complete conformational transition cycle for EfpA. Together, our results provide a structural and mechanistic foundation to comprehend EfpA function and develop EfpA-targeting anti-TB drugs.

## Introduction

Tuberculosis (TB) is a major global infectious disease mainly caused by *Mycobacterium tuberculosis* (*Mtb*). It is estimated that over 10 million new cases of TB occur worldwide and causing more than 1.6 million deaths annually^1^. This made TB the second leading cause of death from a single infectious agent, following COVID-19, in 2021. Over recent decades, the emergence and continuous increase in drug-resistant and multidrug-resistant TB cases have made the global TB epidemic more severe and urgent^1,2^. One of the major mechanisms that causes *Mtb* drug resistance is genetic mutations in drug target-related genes, which render drugs ineffective^3^. In cases of isoniazid (INH), one of the most efficient drugs against *Mtb*, mutations in katG, a catalase-peroxidase responsible for the conversion of isoniazid to its active form, and target enzyme InhA were found^4,5^. Another mechanism of drug resistance is the activation and overexpression of efflux pumps, which reduce the effective drug concentration inside the cell^6^. In some drug-resistant clinical strains, genetic mutations have not been found in known drug targets, but multiple efflux pumps, including EfpA and MmpL7, have been observed with significant overexpression^7–10^. This suggests that these efflux pumps may contribute to drug resistance.

Efflux pumps are widely present in bacteria and can be classified into 5 families: the ATP-binding cassette (ABC) superfamily, the major facilitator superfamily (MFS), multidrug and toxic compound extrusion (MATE) family, resistance-nodulation-division (RND) family, and small multidrug resistance (SMR) family^11^. EfpA, a member of the QacA transporter family in the MFS superfamily^12^, was found to have higher expression level in *Mtb* upon exposure to INH, as demonstrated by DNA microarray^13^. RT‒qPCR analysis showed that EfpA was significantly upregulated in various clinical *Mtb* strains, including strains resistant to INH, rifampicin (RIF), ofloxacin (OFX), or multidrug^14–16^. Overexpression of EfpA from *Mtb* or *M. bovis* BCG in *M. smegmatis* (*Ms*) led to a significant increase in resistance to INH, RIF and amikacin by ten to hundreds of fold, and the resistance was further strengthened with a longer induction time^17^. Furthermore, a mutation of E520V was identified in *Mt*EfpA of an INH-resistant strain but not in any of the susceptible clinical isolates^18^. These suggest EfpA plays a crucial role in drug resistance by effluxing multiple drugs in *Mtb*. Moreover, high-density mutagenesis and deep-sequencing studies revealed that EfpA is essential for *Mtb* growth in vitro, indicating that it may also efflux critical endogenous substrates^19,20^. However, the identity of the endogenous substrates of EfpA remains elusive.

The essential role of EfpA in *Mtb* and its exclusive presence in Actinomycetes make EfpA an attractive and promising drug target. Recently, new classes of *Mtb* inhibitors, the BRD-8000 series (BRD-8000s) and BRD-9327, were identified through large-scale chemical-genetics screening and have been demonstrated to specifically target EfpA^21,22^. Among them, the optimized inhibitor BRD-8000.3 exhibited the highest inhibitory activity against *Mtb* (MIC90 = 800 nM). BRD-8000.3 is effective against non-replicating, phenotypically drug-tolerant *Mtb* and *Mycobacterium marinum* (*Mmar*), but not *Ms*^21,23^. BRD-8000s exhibited non-competitive inhibition of the efflux of a known EfpA substrate, ethidium bromide (EtBr)^24^. Furthermore, expressing BRD-8000s-resistant *Mt*EfpA (V319) or *Mt*EfpA (WT) mutants in *Mtb* does not affect the sensitivity of *Mtb* to INH^21^, which is mediated by EfpA efflux^10,16^. This suggests that BRD-8000s kill *Mtb* by inhibiting the endogenous function of *Mt*EfpA. Although oral administration of BRD-8000.3 showed good plasma exposure in mice and a low risk of drug‒drug interactions^21^, further optimization is needed to enhance its inhibitory activity and improve its physicochemical properties for its use as a drug against *Mtb*. However, the binding site and inhibition mechanism of BRD-8000.3 on *Mt*EfpA are not yet fully understood.

In this study, we expressed and purified *Mt*EfpA and *Ms*EfpA and determined their cryo-EM structures in an outward-open conformation. In the *Mt*EfpA structure, three lipids are sequentially bound in head-to-head and tail-to-tail arrangements within a channel that traverses from the inner leaflet through the central pocket to the outer leaflet of the phospholipid bilayer. The cryo-EM structure of *Mt*EfpA complex with BRD-8000.3 shows that BRD-8000.3 occupies the lipid binding pocket near the inner leaflet of the lipid bilayer, rather than the central pocket, suggesting that BRD-8000s competitively inhibit lipid binding to EfpA but non-competitively inhibit drug efflux. Finally, by comparing our structures with the inward-open structure of EfpA predicted by AlphaFold^25^, we proposed a “staircase-flips” model for the transport of lipids by EfpA. Our results provide structural insight into the endogenous function of EfpA and the mechanism of its inhibitors to help future development of anti-*Mtb* drugs.

## Results

### EfpA is in an outward-open conformation

**Extended Data Fig. 1 |:**
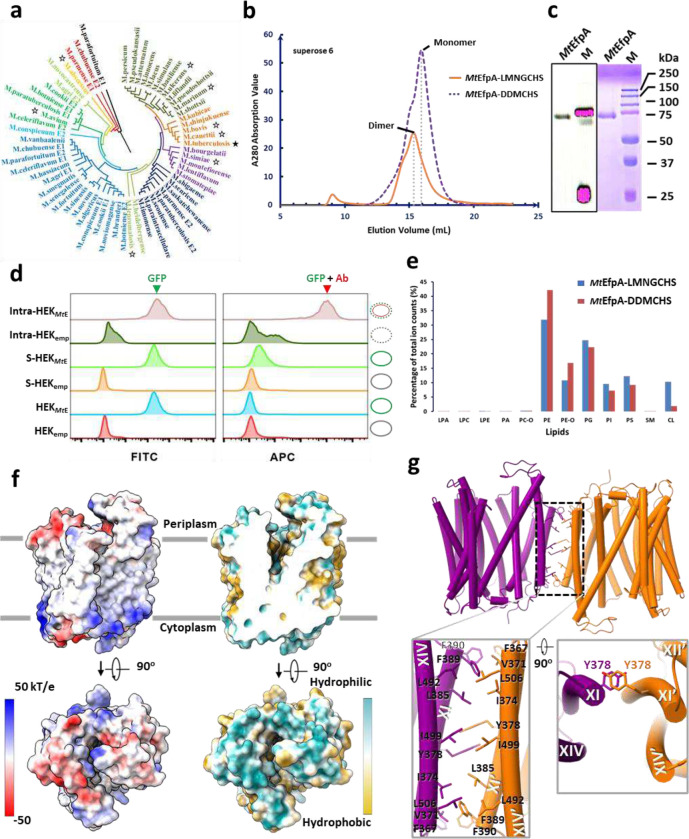
Characterization and structural analysis of EfpA. **a**, Phylogenetic tree of EfpA homologues. *M. tuberculosis* and other pathogenic species of mycobacterium are highlighted with black star and empty stars, respectively. **b**, SEC profile of *Mt*EfpA with C-terminal GFP tag after solubilized by LMNG-CHS. **c**, SDS-PAGE with GFP fluorescence scan (left) and Coomassie stain (right). **d**, Flow cytometry assay of HEK w/wo *Mt*EfpA-GFP expressed (HEK_emp_/HEK_*Mt*E_) and incubated with APC anti-GFP antibody before/after permeabilization (S/Intra). **e**, Lipidomics and small-molecule analysis of dimeric *Mt*EfpA-LMNGCHS and monomeric *Mt*EfpA-DDMCHS. **f**, Electrostatic surface and hydrophobic surface of *Mt*EfpA. **g**, Dimeric interface of *Mt*EfpA dimer.

EfpA is widely conserved among mycobacterial species, with some species having multiple paralogs^17^. Multiple sequence alignment shows high homology (>50%) of EfpA homologs across different species, including both pathogenic and nonpathogenic *Mycobacterium* (Extended Data Fig. 1a). Specifically, *Mt*EfpA and *Ms*EfpA show a remarkable 79% sequence identity. To express *Mt*EfpA with a C-terminal GFP and strep tag, a codon-optimized gene was integrated into the Expi293F cell genome using sleeping beauty transposase^26^, and stable cell lines with high expression were screened by flow cytometry. The protein purified using affinity and size-exclusion chromatography (Extended Data Fig. 1, b and c) was used for single-particle cryo-EM study.

**Extended Data Fig. 2 |:**
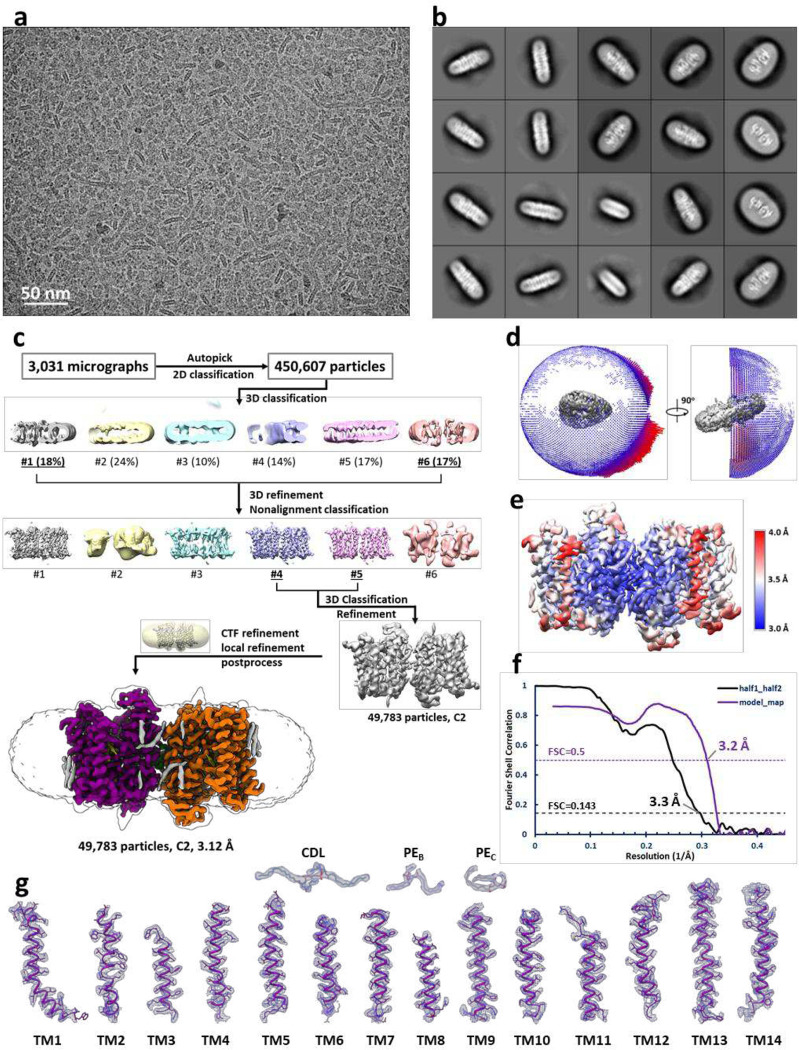
Cryo-EM reconstruction of dimeric *Mt*EfpA. **a**, Representative cryo-EM micrograph of *Mt*EpfA. **b**, 2D class averages of *Mt*EfpA with a box size of 266 Å. **c**, Flowchart for processing cryo-EM data of *Mt*EpfA. **d**, Particle angle distribution for the final map of dimeric *Mt*EfpA. **e**, Local resolution of the final cryo-EM map. **f**, Gold standard Fourier shell correlation (FSC) curve for the final reconstruction. Half map #1 vs. half map #2 of *Mt*EfpA with masked shown in black. Model vs. refined map with masked shown in purple. **g**, Close-up view of all structure elements fitted in densities with contour level at 5σ (blue).

**Extended Data Fig. 3 |:**
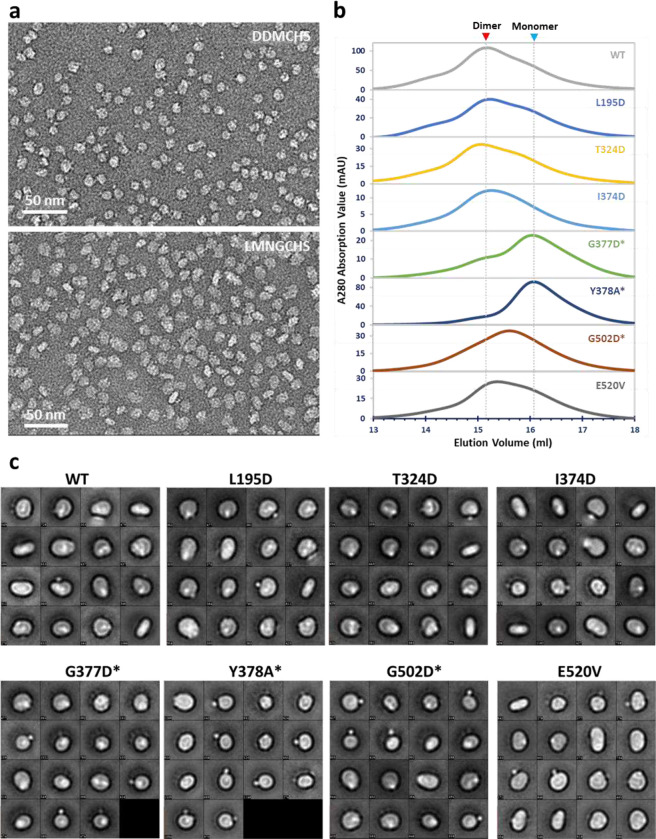
Negative stain analysis of *Mt*EfpA and mutants. **a**, Representative micrographs of monomeric *Mt*EfpA purified with DDMCHS (upper) and dimeric *Mt*EfpA purified with LMNGCHS (bottom). **b**, Size exclusion chromatography (SEC) profile of *Mt*EfpA wide type and mutants purified with LMNGCHS. The mutants forming monomers were marked with stars. **c**, Negative stain 2D averages of *Mt*EfpA wide type and mutants purified with LMNGCHS.

**Fig. 1 |:**
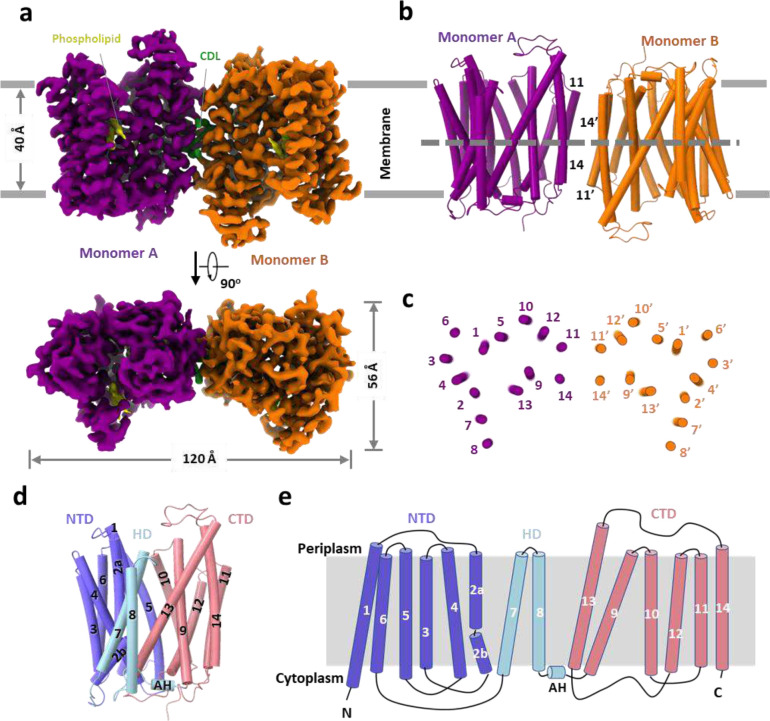
Overall structure of MtEfpA. **a**, 3D reconstruction map of dimeric *Mt*EfpA in sideview (top) and topview (bottom). Two *Mt*EfpA monomers are colored purple and orange. Phospholipids are colored yellow, and cardiolipin (CDL) is in green. **b**, Carton model of *Mt*EfpA dimer shown as in (**a**). **c**, Cross sectional view of *Mt*EfpA at the level indicated as the dotted line in (**b**). **d**, Structure of monomeric *Mt*EfpA. The hinge domain TM7–8 (light blue) connects the NTD domain TM1–6 (Blue) and CTD domain TM9–14 (pink). The second transmembrane fragment break into two half helixes (2a and 2b). An amphiphilic helix (AH) between TM8 and TM9, locates on the cytoplasm surface of membrane. **e**, Topology schematic of *Mt*EfpA.

The cryo-EM map of *Mt*EfpA at 3.1-Å resolution revealed a dimeric assembly with a total of 28 transmembrane segments (TMs) in the micelles, as well as two loops and two terminal tails extending out of the micelles (Fig. 1a and Extended Data Fig. 2c). The well-resolved TMs with clear α-helical and sidechain features allowed us to build a model of *Mt*EfpA from residues 45 to 519 (Extended Data Fig. 2g). The EfpA dimer is in an antiparallel configuration, with TM11 and TM14 mediating the interactions between the two monomers (Fig. 1, b and c). These interactions are mainly hydrophobic, except for a pi-bond formed by two Y378 residues from each monomer (Extended Data Fig. 1g). Mutation of Y378 to alanine disrupts dimer formation (Extended Data Fig. 3, b and c). To determine the topology of *Mt*EfpA in the membrane, we incubated *Mt*EfpA-cGFP expressing cells, before and after permeabilization, with an allophycocyanin (APC) conjugated anti-GFP antibody, and then analyzed GFP and APC signals using flow cytometry. APC signals were observed in permeabilized cells, but not in nonpermeabilized cells (Extended Data Fig. 1d). This indicates that the C-terminus of *Mt*EfpA in the membrane is inside the cytosol. Therefore, the antiparallel *Mt*EfpA dimer was likely formed during solubilization and purification. Interestingly, a similar phenomenon has also been reported in other MFS transporters^27^.

In each monomer, *Mt*EfpA contains 14 transmembrane helices (TMs 1–14) (Fig. 1d), instead of 12 TMSs in canonical MFS transporters^28^, with both termini located on the cytoplasmic side (Fig. 1e). Different from canonical MFS fold, which comprises two domains with pseudo-two-fold symmetry connected by a long loop^27,29–32^, *Mt*EfpA has a hinge domain (HD), which contains a long loop (229–239), two TMs (TM7–8), and an amphiphilic helix (AH), to connect the N-terminal domain (NTD) consisting of TMs 1–6 and the C-terminal domain (CTD) consisting of TMs 9–14 (Fig. 1, d and e). Furthermore, the root-mean-square deviation (RMSD) value between NTD and CTD is 11.5 Å for 96 Cα atoms, indicating a large structural difference and lack of internal pseudo-two-fold symmetry in EfpA.

**Extended Data Fig. 4 |:**
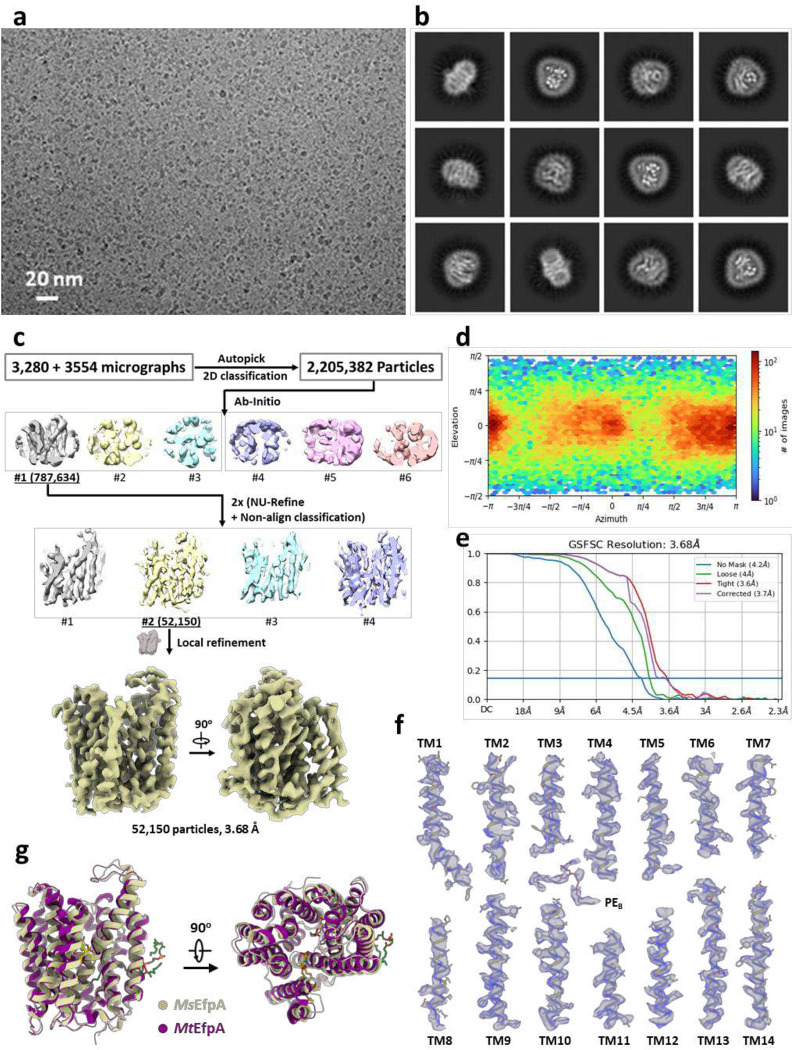
Cryo-EM reconstruction of monomeric *Ms*EfpA. **a**, Representative cryo-EM micrograph of *Ms*EpfA. **b**, 2D class averages of *Ms*EfpA with a box size of 180.4 Å. **c**, Flowchart for processing cryo-EM data of *Ms*EpfA. **d**, Particle angle distribution for the final map of monomeric *Ms*EfpA. **e**, Gold standard Fourier shell correlation (FSC) curve for the final reconstruction. **f**, Close-up view of all helixes (olive) fitted in densities with contour level at 5σ (blue) and a molecular of PE (brown) fitted into the density at lipid binding site B. **g**, Superimposing the model of *Ms*EfpA (olive) to *Mt*EfpA (purple).

Unlike the formation of dimeric *Mt*EfpA resulting from solubilizing membrane with LMNG-CHS, DDM-CHS generated predominantly monomeric EfpA (Extended Data Figs. 1b and 3a). To investigate the conformation of monomeric EfpA, we determined the structure of *Ms*EfpA in DDM-CHS at 3.7-Å resolution (Extended Data Fig. 4). The structure of monomeric *Ms*EfpA is essentially identical to *Mt*EfpA, with an RMSD value of 0.868 Å for 425 Cα atoms (Extended Data Fig. 4g). Thus, both structures of dimeric *Mt*EfpA and monomeric *Ms*EfpA are in the outward-open conformation (Extended Data Figs. 1f and 4g).

### Three lipids within EfpA define an internal pathway

**Extended Data Fig. 5 |:**
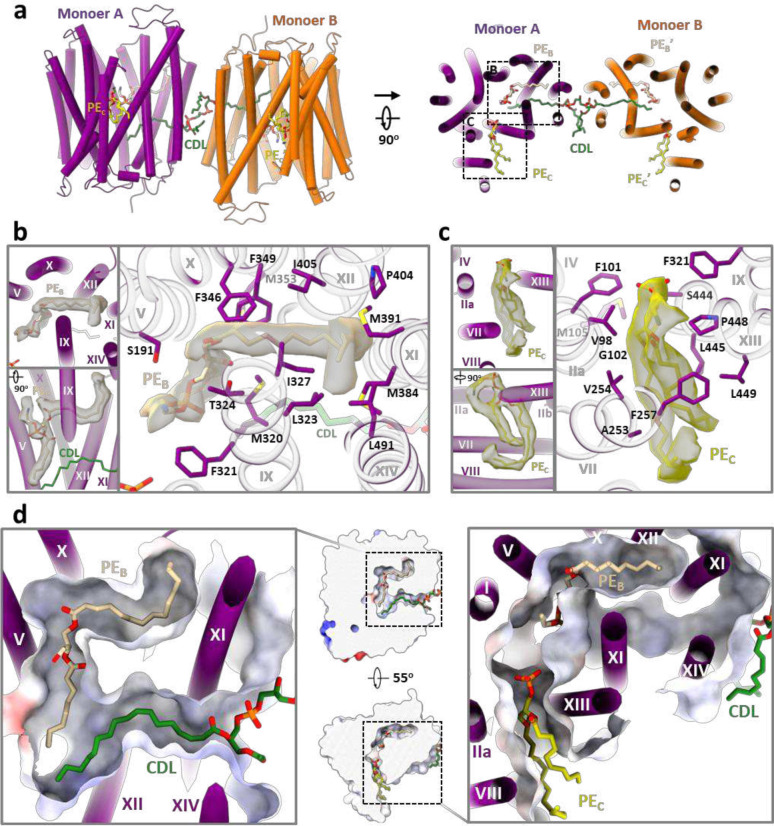
Lipids binding channel in *Mt*EfpA. **a**, The cartoon model of *Mt*EfpA (purple and orange) binding with cardiolipin (green) and two PE (wheat and yellow) from side view (left) and top view (right). **b**, Superimposition of phosphatidylethanolamine (PE_B_) and the density at site B at a contour level of 6σ, presented in top view (left upper), side view (left bottom), and the *Mt*EfpA residues interacting with PE_B_ within 4 Å (right). **c**, The density at site C (6σ) fitted with the model of phosphatidylethanolamine (PE_C_) showed in top view (left upper), side view (left bottom), and the residues of *Mt*EfpA interacted with PE_c_ within 4 Å (right). **d**, Cross-section views of lipids binding cavities in *Mt*EfpA. Left, the cavities of cardiolipin (CDL, green) and phosphatidylethanolamine (PE_B_, wheat). Right, the cavities of phosphatidylethanolamines (PE_B_ and PE_C_).

**Fig. 2 |:**
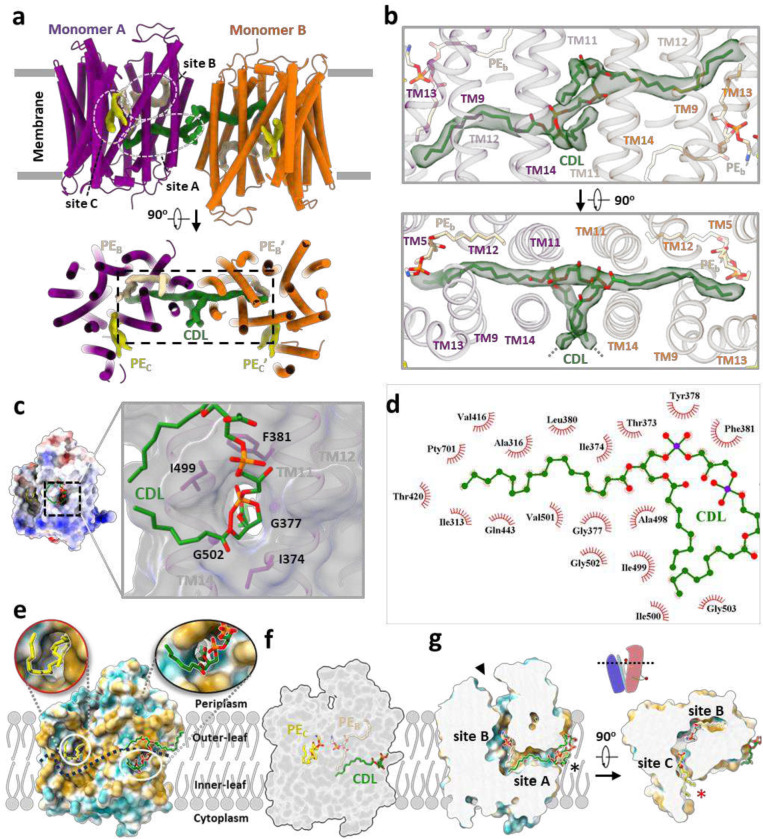
Three lipids binding in a channel in *Mt*EfpA. **a**, The model of *Mt*EfpA with densities of cardiolipin (CDL, green) and 2 molecular of Phosphatidylethanolamine (PE, colored wheat at site B and yellow at site C). **b**, Superimposition of the *Mt*EfpA model and the cardiolipin density (5σ) at site A, shown in side view (upper) and top view (bottom). The gray dashed lines represent the missing ends of two tails extending inward toward the membrane. **c**, The surface and residues at the lateral gate of cardiolipin insert into *Mt*EfpA. **d**, The residues of *Mt*EfpA that interact with cardiolipin within 4 Å. **e**, the two lateral gates of a channel in *Mt*EfpA open separately towards the inner and outer leaflets of the membrane. The dashed line represents the boundary between the inner leaflet and outer leaflet of membrane. **f**, Three lipids bind to *Mt*EfpA in “Z”-shape tunnel from a side view. **g**, Cross-sectional perspectives of the lipids-binding channel in *Mt*EfpA. Left, view from side. Right, view from top. The two lateral gates are indicated by black and red stars, while the funnel opens toward the periplasm, marked with a black triangle. The cartoon on top represents the cross-sectional position from a lateral perspective.

In the cryo-EM map of dimeric *Mt*EfpA, strong nonproteineous densities were identified at three adjacent sites (sites A-C) within *Mt*EfpA (Fig. 2a). These densities exhibit two or four elongated tails which do not correspond to the shapes of detergent molecules (Fig. 2a and Extended Data Fig. 5, b and c). In addition, the density at site A with four tails has two long tails inserted into the hydrophobic pockets of each *Mt*EfpA monomer (Fig. 2b). These features suggest that the additional densities are lipids. Untargeted lipidomic analysis showed that the amount of cardiolipin in *Mt*EfpA dimer is four times higher than that in monomeric *Mt*EfpA purified with DDM-CHS (Extended Data Fig. 1e). Therefore, the four-tail density linking two monomers is likely cardiolipin which indeed fits well into this density (Fig. 2b). The other two lipids are likely glycerophospholipids, although the resolution is not sufficient to confidently identify the headgroups. Nevertheless, phosphatidylethanolamine (PE), the most abundant lipid identified by lipidomics in dimeric *Mt*EfpA, fits well into each of these two densities and was used in atomic model building (Extended Data Fig. 5, b and c). Molecular dynamic simulation was utilized to analyze the binding mode and stability of these cardiolipin and PE molecules bound to *Mt*EfpA, showing stable binding of cardiolipin in site A pocket for up to 500 ns (RMSD < 4.0 Å) and more motion for PE at site B (PE_B_, RMSD < 10.0 Å) and PE at site C (PE_C_, RMSD < 7.0 Å) (Extended Data Fig. 11a).

Within our cryo-EM structure of *Mt*EfpA, the three lipids are generally parallel, instead of perpendicular, to the membrane surface. The cardiolipin molecule has its hydrophilic head group at the dimer interface, two acyl chains inserted into the crevice formed by TM11-TM12 and TM9-TM14 of each monomer, and the other two acyl chains extending into the membrane (Fig. 2b, bottom). The residues I374, G377, F381, I499, and G502 at the dimeric interface of EfpA formed a lateral gate to accommodate the insertion of the hydrophobic cardiolipin acyl chain (Fig. 2c). Single mutations of G377D and G502D, which are located at the lateral gate of cardiolipin acyl chains, weaken EfpA dimer formation (Extended Data Fig. 3, b and c). However, mutations of L195D and T324D, situated at lipid-binding site B, exhibited no discernible impact on dimer formation. The hydrophobic tails of cardiolipin that insert into EfpA interact with I313, A316, V501, A498, T373, I412, V416, T420, and Q443 from TM9, TM14, TM11, TM12, and TM13 (Fig. 2, b and d). The lipid at site B (PE_B_), adjacent to cardiolipin, is surrounded by TM9, TM14, TM11, TM12, TM10, and TM5, with its hydrophobic acyl chains interacting with M320, L323, I327, L491, M384, P404, I405, F349, and F346 (Extended Data Fig. 5b). The lipid at site C (PE_C_) binds in the gap between the hinge domain and TM9-TM13 from CTD, with its acyl chains extending into the membrane (Extended Data Fig. 5c). Conservation analysis using ConSurf^33^ on EfpA from different species of *Mycobacterium* revealed that lipid-binding site A exhibits variability, while sites B and C are highly conserved. (Extended Data Fig. 10). In addition, a hydrophilic cavity is located at the center of *Mt*EfpA, formed by the head groups of PE_B_ and PE_C_, as well as TM1, TM4, TM2a, TM13 and TM5. The central pocket exhibits a width of approximately 6–10 Å and possesses a negatively charged inner surface with residues Y97, S155, S69, T66, Q62, S191, Q443, and S444 (Extended Data Fig. 10b). Among these residues, Y97, which is solvent exposed and can undergo protonation under neutral and alkaline conditions while accepting protons under acidic conditions, is conserved in all species. This suggests that Y97 may trigger the conformational transition of EfpA through protonation and deprotonation^31^.

A solvent-accessible pathway within *Mt*EfpA was identified using the MOLEonline program^34^, consisting of three openings oriented toward the periplasm, the inner and outer leaflets of the phospholipid bilayer (Fig. 2e and Fig. 4e, right). Intriguingly, this pathway accommodates the sequential binding of three lipids, forming a “Z”-shaped pathway that starts from the inner leaflet, traverses the central pocket, and reaches the outer leaflet of the phospholipid bilayer (Fig. 2, f and g). The lipids bind within the pathway in a head-to-head and tail-to-tail arrangement (Fig. 2g, Extended Data Fig. 5d, and Supplementary Video 1).

### BRD inhibitors occupy lipid binding sites in EfpA

**Extended Data Fig. 6 |:**
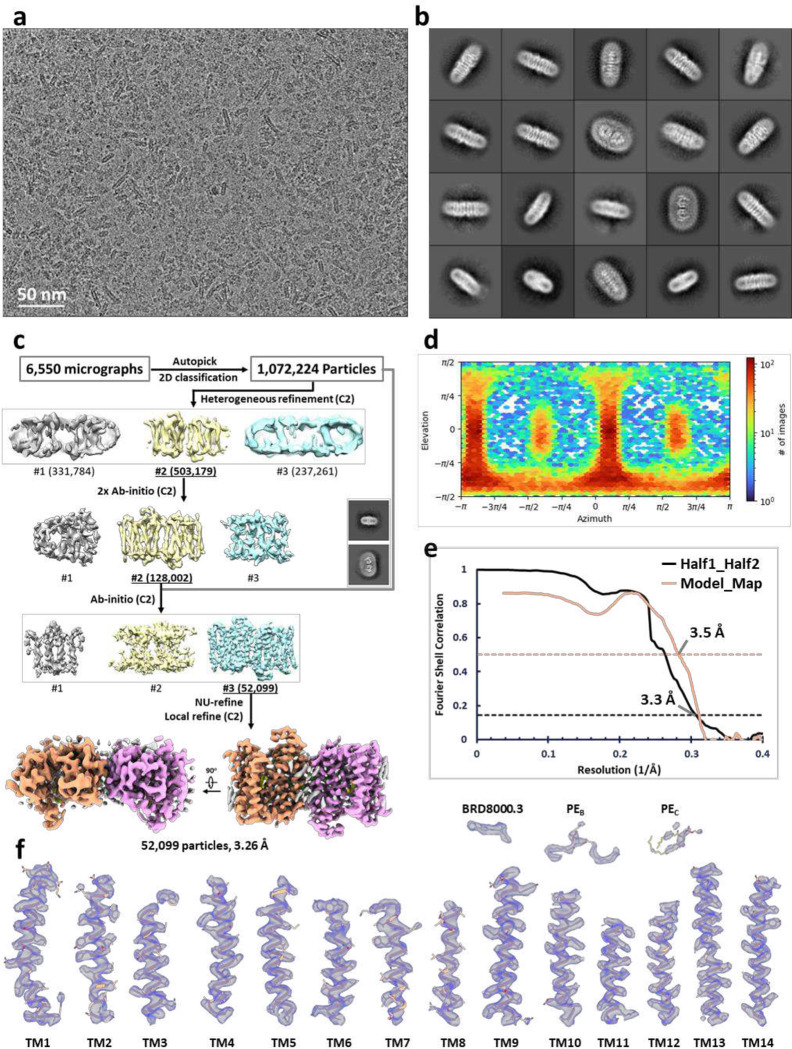
Cryo-EM reconstruction of dimeric *Mt*EfpA-BRD8000.3. **a**, Representative cryo-EM micrograph of *Mt*EpfA complexed with BRD-8000.3. **b**, 2D class averages of *Mt*EfpA with a box size of 252 Å. **c**, Flowchart for processing cryo-EM data of *Mt*EpfA-BRD8000.3. **d**, Particle angle distribution for the final map of dimeric *Mt*EfpA-BRD8000.3. **e**, Gold standard Fourier shell correlation (FSC) curve for the final reconstruction of *Mt*EfpA-BRD8000.3. **f**, Close-up view of all structure elements of *Mt*EfpA-BRD8000.3 fitted in densities with contour level at 5σ (blue).

**Extended Data Fig. 7 |:**
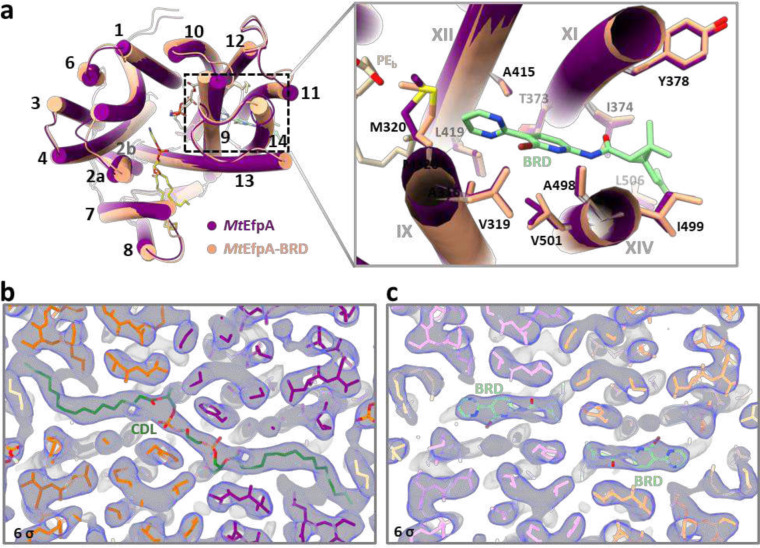
Structural comparison of *Mt*EfpA binding with cardiolipin and BRD-8000.3. **a**, Superimposing structure of *Mt*EfpA (purple) and *Mt*EfpA-BRD (orange) at binding site A. **b, c**, Density of cardiolipin (dark green) in *Mt*EfpA (**b**) and BRD-8000.3 (light green) in *Mt*EfpA-BRD (**c**) with contour level at 6σ.

**Fig. 3 |:**
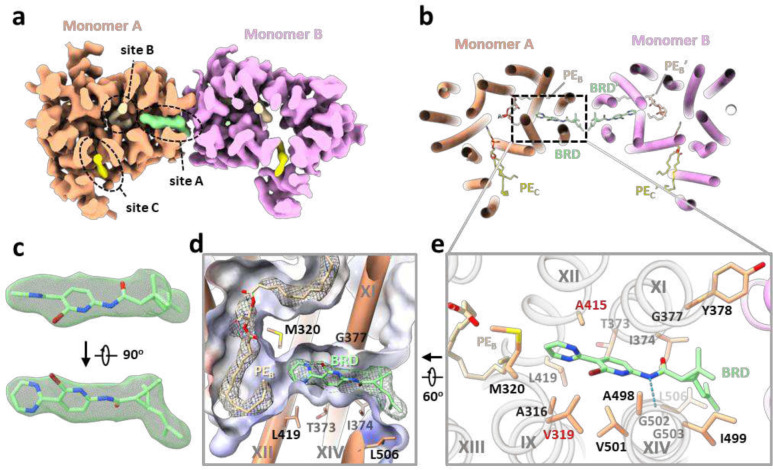
Structure of *Mt*EfpA bound to the inhibitor BRD-8000.3. **a**, Top view of BRD-8000.3 density (light green) at the lipid binding site A in one *Mt*EfpA monomer (light orange). **b**, Cartoon model of *Mt*EfpA bound to BRD-8000.3 (BRD, light green) and two phosphatidylethanolamine (PE) molecules (wheat at site B and yellow at site C). **c**, Superimposition of the BRD-8000.3 model and density (5σ). **d**, Electrostatic surface of the BRD-8000.3 binding pocket. **e**, Residues of *Mt*EfpA (light orange) interact with BRD-8000.3 (light green) within 4 Å. The hydrogen bond between A489’s O3 of *Mt*EfpA and N9 of BRD-8000.3 is shown in a blue dashed line. Resistant mutation residues are labeled in red.

Two series of EfpA-targeting *Mtb* inhibitors, BRD-8000s and BRD-9327, were recently identified through high-throughput chemical-genetic screening^21,22^. These two series of compounds inhibit EfpA efflux in a non-competitive manner, and display potentiation when used in combination^22^. To reveal their inhibitory mechanism, we supplemented BRD-8000.3, which exhibited the highest inhibitory activity, during *Mt*EfpA purification and determined the cryo-EM structure of *Mt*EfpA-BRD8000.3 complex at 3.3-Å resolution (Extended Data Fig. 6). In this cryo-EM map, BRD-8000.3 fits well into the density at lipid-binding site A, displacing the cardiolipin observed in our cryo-EM map of *Mt*EfpA without compound (Fig. 3, a and c). The density of BRD-8000.3 is distinct from that of cardiolipin (Extended Data Fig. 7, b and c). Molecular dynamic simulations show that BRD-8000.3 can stably bind to the site A pocket for up to 500 ns with an RMSD within 2 Å (Extended Data Fig. 11b).

The binding of BRD-8000.3 to *Mt*EfpA caused only minor structural changes, as compared to the structure of *Mt*EfpA without compound (RMSD = 0.462 Å for 475 Cα atoms) (Extended Data Fig. 7a). Similar to the hydrophobic tail of cardiolipin inserted in *Mt*EfpA, BRD-8000.3 binds in the gap between TM11-TM12 and TM9-TM14, interacting with M320, A316 and V319 on TM9, V501, A498, I499, G502, G503 and L506 on TM14, T373, I374, G377, Y378 on TM11, and A415 on TM12 (Fig. 3, b and e). A previous study identified several BRD-8000.3 resistant mutants, including V319F in *Mt*EfpA and V319M, A415V in *Mm*EfpA, which has 90% identity to *Mt*EfpA and is same at these residues^22^, corroborating our structural finding of BRD-8000.3 bound in site A (Fig. 3e and Extended Data Fig. 11c). The large side chains resulting from these mutations likely cause steric hindrance with BRD-8000.3 and prevent its binding to *Mt*EfpA. In addition to hydrophobic interactions, a hydrogen bond between N9 of BRD-8000.3 and O3 of *Mt*EfpA A498 may facilitate the binding of BRD-8000.3 and displacement of lipid from the site A pocket (Fig. 3e).

In contrast, the *Mm*EfpA mutants resistant to BRD-9327 were identified with mutations G328C, G328D, F346L, and A339T^22^, which are located at lipid-binding site B (Extended Data Fig. 11d, left). AutoDock Vina^35^ shows that BRD-9327 can fit into the site B pocket, thus preventing lipid binding (Extended Data Fig. 11d, right). BRD-9327 and BRD-8000s binding to distinct sites in EfpA is consistent with the reported synergistic inhibition of *M. marinum* when combining BRD-8000.3 and BRD-9327^22^. Thus, our structural analyses indicate that BRD-8000s and BRD-9327 compete with lipids for binding to two distinct sites in EfpA, thus inhibiting the endogenous functions of EfpA and ultimately leading to the death of *Mycobacterium*.

### EfpA resembles related lipid transporters

The presence of a lipid-occupying pathway within EfpA and the displacement of these lipids by small-molecule *Mtb* inhibitors imply a connection between these lipids and EfpA’s essential functions for *Mtb*. To gain further insights into EfpA function, we conducted structure-based functional predictions using COFACTOR^36^ against BioLip protein function database and Dali server^37^ against the Protein Data Bank (PDB). Both searches consistently identified MFSD2A, a lysophospholipid transporter in the MFS superfamily, as the top hit. MFSD2A transports long-chain unsaturated fatty acids with a zwitterionic lysophosphatidylcholine headgroup, such as docosahexaenoic acid (DHA) and α-linolenic acid (ALA), across the blood brain barrier^38,39^. MFSD2A is the best studied MFS lipid transporter with well-resolved substrate lipid densities and high-resolution structures in both outward- and inward-open conformations^38–42^.

**Extended Data Fig. 8 |:**
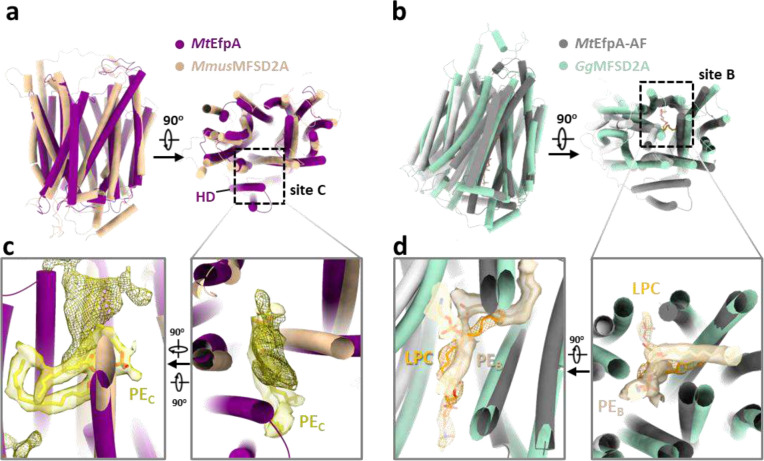
Comparison of the structures of *Mt*EfpA to the structures of MFSD2A. **a**, Superimposing the outward-open structure of *Mt*EfpA (purple) with the outward-open structure of lysophospholipid transporter MFSD2A from *Mus musculus* (*Mmus*MFSD2A, light orange. PDB ID: 7N98). **b**, Superimposing the inward structure of *Mt*EfpA-AF (gray) with the inward structure of MFSD2A from *Gallus gallus* (*Gg*MFSD2A, blue-green. PDB ID: 7MJS). **c**, Zoomed-in view of lipid binding site C shows the lipids density in *Mt*EfpA (yellow) and *Mmus*MFSD2A (mesh, EMDB-24252). **d**, Zoomed-in view of lipid binding site B shows the lipids density in *Mt*EfpA (wheat) and the density of lysophosphatidylcholine (LPC, orange, EMDB-23883) in *Gg*MFSD2A.

Upon superimposing our structure of *Mt*EfpA and the outward-open structure of MFSD2A from *Mus musculus* (*Mmus*MFSD2A)^41^, we found that, despite low sequence homology (11%), they exhibit high-level structural similarity, except for the additional hinge domain of *Mt*EfpA (Extended Data Fig. 8a). Furthermore, the density of the substrate, lysophospholipid, in the outward-open conformation of *Mmus*MFSD2A corresponds to the lipid bound to site C in *Mt*EfpA, albeit with a more vertical orientation (Extended Data Fig. 8c). Additionally, we compared the AlphaFold-predicted structure of *Mt*EfpA, which adopts an inward-open conformation, with the recently reported inward-open structure of MFSD2A from *Gallus gallus* (*Gg*MFSD2A)^40^. Similar to their outward-open counterparts, the inward-open conformations of *Mt*EfpA and *Gg*MFSD2A also exhibit analogous folding patterns (Extended Data Fig. 8b). Moreover, in the inward-open conformation of *Gg*MFSD2A, the substrate lysophosphatidylcholine (LPC) is situated in a pocket corresponding to site B in *Mt*EfpA (Extended Data Fig. 8d). The remarkable structural similarity in both inward and outward conformations between MFSD2A and *Mt*EfpA, together with their highly analogous lipid-binding sites, strongly supports the notion that *Mt*EfpA shares a similar function with MFSD2A as a lipid flippase, facilitating the translocation of substrate lipids between the inner and outer leaflets of the *Mtb* membrane.

### Conformational transition of EfpA during substrate efflux

**Extended Data Fig. 9 |:**
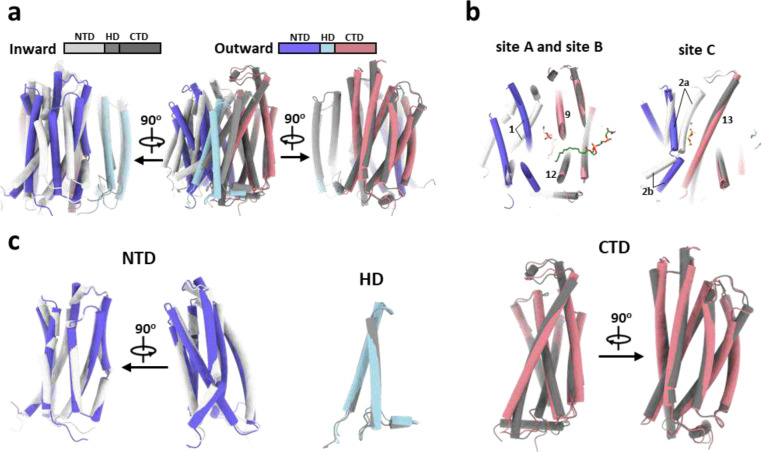
Inward and outward state of *Mt*EfpA. **a**, Superimposing AlphaFold-predicted inward-state of *Mt*EfpA (*Mt*EfpA-AF, lightgray-gray-darkgray) with outward state of *Mt*EfpA (blue-lightblue-lightpink). **b**, The lipid binding site A/B (left) and site C (right) after superimposing. **c**, Alignment of NTD (left), HD (mid) and CTD (right) from *Mt*EfpA and *Mt*EfpA-AF separately.

**Fig. 4 |:**
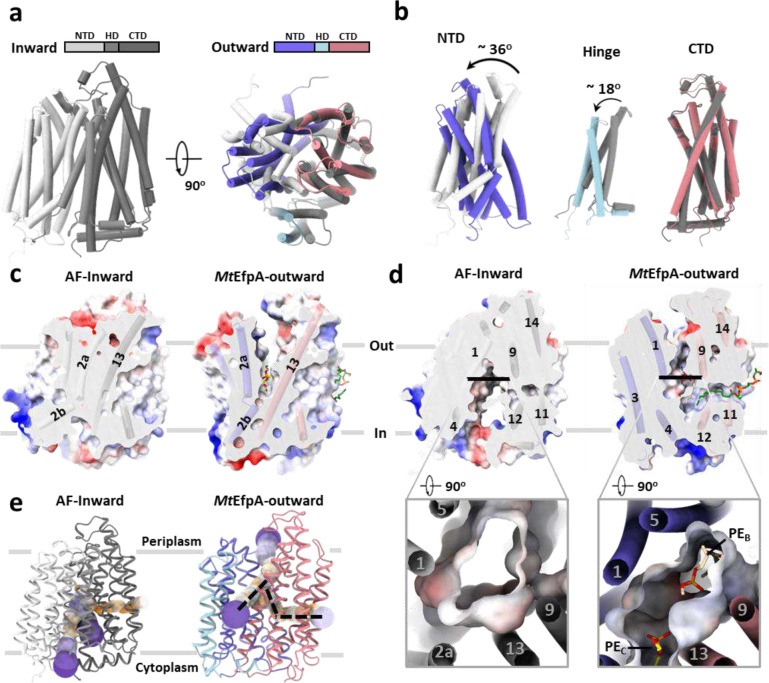
Comparison of inward and outward facing state of *Mt*EfpA. **a**, Superimposition of inward state of *Mt*EfpA (lightgray-gray-darkgray), predicted by AlphaFold, with outward state of *Mt*EfpA (blue-lightblue-lightpink). **b**, Movement of *Mt*EfpA’s NTD and hinge domain from an inward state to an outward state. **c**, The PE binding cavity at site C in outward state (right) was blocked by TM2a moving near TM13 in inward state (left). **d**, The binding cavity at site B changes from inward open (left) to outward open (right). **e**, The tunnel in inward and outward state of *Mt*EfpA.

The structures of EfpA from different *Mycobacterium* species were predicted using AlphaFold^25^, and all of them, including *Mt*EfpA, exhibited an inward-open conformation (Fig. 4a and Extended Data Fig. 9a). Compared to the outward-open conformation of our *Mt*EfpA structures, the CTD in the predicted inward-open structure exhibits minimal variation (Fig. 4b, right), while the NTD and HD of the inward-open conformation rotate by approximately 36° and 18°, respectively (Fig. 4b, left and middle). In the inward-open conformation, the rotation of HD and the movement of TM2a toward TM13 result in the occlusion of lipid-binding site C (Fig. 4c). Additionally, the opening of the central hydrophilic pocket shifts from the cytoplasm to the periplasm (Fig. 4d). Despite the rotations and movements, the structures of individual domains of EfpA remain largely unchanged between the outward- and inward-open conformations (Extended Data Fig. 9c). This indicates that EfpA may employ the canonical rocker-switch mechanism^43^ for the transport of hydrophilic drugs bound at the central pocket (Supplementary Video 2).

The solvent-accessible pathway in the inward-open conformation of EfpA differs from that in the outward-open conformation (Fig. 4e). The pathway in the inward-open conformation constricts at lipid-binding site A, and the lateral gate opens to the middle of membrane, while the other two funnels of the pathway open toward the cytoplasm and the inner leaflet of membrane (Fig. 4e left). These observations suggest a mechanism for EfpA to efflux the drug molecules from the cytoplasm: the compounds bind to the central hydrophilic pocket of EfpA in its inward-open conformation and are subsequently released to the periplasm when EfpA transitions to the outward-open conformation. Such conformational changes may also cause the association and dissociation of lipid molecules, as well as the movement of lipids between different binding sites within EfpA.

## Discussion

EfpA was the first identified efflux pump in *Mtb*^12^ and has been implicated in multidrug resistance, particularly against INH^10,14–17^. The essential role of EfpA in the in vitro growth of *Mtb*^19,20^ indicates that, in addition to export drugs, EfpA may have other crucial, unknown endogenous functions. In this study, the presence of a lipid-binding tunnel in *Mt*EfpA that spans from the inner leaflet to the outer leaflet of the membrane, along with inhibitors targeting the lipid-binding sites and the significant structural and lipid-binding site similarity to the lysophospholipid transporter MFSD2A, strongly suggest that EfpA functions as a lipid transporter.

**Fig. 5 |:**
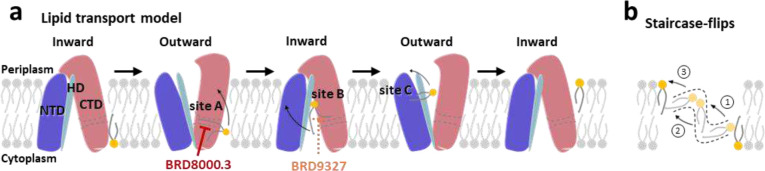
Proposed model for lipid transport by *Mt*EfpA and small-molecule inhibition. **a**, Lipid transport model for *Mt*EfpA and distinct target sites of different inhibitors. NTD, N-terminal domain. HD, hinge domain. CTD, C-terminal domain. The substrate lipid flips to adjacent binding site or releases to the outer-leaf of membrane during each conformational transition. BRD-8000.3 targets lipid-binding site A and BRD-9327 was supposed to target lipid-binding site B to inhibit *Mt*EfpA function. **b**, Lipids undergo a stepwise flipping, resembling a staircase, from the inner leaflet to the outer leaflet of the membrane.

Two general models have been proposed to describe lipid translocation by lipid transporters: the trap-and-flip model^44,45^ and the credit-card model^46–48^. While the only characterized structures of MFS lipid transporters, MFSD2A^40,41^ and LtaA^49^, suggest the possibility of MFS lipid transporters employing the rocker-switch^42,45^ or trap-and-flip^45^ mechanism, the rotational dynamics of lipids within the transmembrane region remain unclear. Based on the lipid-binding pathway observed in *Mt*EfpA, we propose a “staircase-flips” model to elucidate how EfpA translocate lipids from the inner membrane leaflet to the outer leaflet (Fig. 5, a and b). In this model, when a proton is released from the central pocket to the intracellular milieu, it triggers the transition of EfpA from an inward-open conformation to an outward-open conformation, expanding lipid-binding site A and facilitating the binding of substrate lipids from the inner-leaf of the membrane. Subsequently, in multiple conformational changes of EfpA, the substrate lipid gradually flips from lipid-binding site A to site B and site C before ultimately being released to the outer-leaf of the membrane (Fig. 5a). Notably, the inhibitor BRD-8000.3 prevents lipid entry into the pathway, while BRD-9327 likely disrupts lipid flipping from site A to site B.

Despite these findings, the exact substrate lipid of *Mt*EfpA remains elusive and requires further investigation. Additionally, although BRD-8000s and BRD-9327 have been reported to competitively inhibit the efflux of ethidium bromide (EtBr), a hydrophilic substrate of EfpA^21,22^, the relationship between the drug efflux and lipid transport functions of EfpA remains unclear. In conclusion, our work sheds light on the endogenous functions of EfpA, elucidates inhibitor mechanisms, and proposes a comprehensive lipid transport model. These findings provide a foundation for the structure-based design and optimization of novel EfpA-targeting drugs against *Mtb*, and advance our understanding of the mechanisms of MFS lipid transporters.

## Methods

### Protein overexpression and purification.

The DNA coding sequences of full-length *Mt*EfpA were optimized for expression in Expi293F and cloned into pSBbi vector with GFP and Strep-Tag II at C-terminal (pSBbi-*Mt*EfpA-CGS)^26^. pSBbi-*Mt*EfpA-CGS was co-transfected with pCMV(CAT)T7-SB100, which contains gene of sleeping beauty transposase, to Expi293F cell line. After a week of screening with 2 mg/L puromycin, a cell line with high expression of *Mt*EfpA was obtained. The cells with *Mt*EfpA were grown at 37 °C with 8% CO2 to 4 × 10^6^ cells per milliliter and harvested after centrifugation at 2,000 rpm for 10 min. Cells pellet were resuspended with buffer A (20 mM Hepes pH 7.0, 300 mM NaCl), then lysed by passing through a microfluidizer (ATS Scientific Inc) at 200 bars after adding protease inhibitor cocktail. Cell debris was removed by centrifugation at 8,000 rpm for 10 min followed by collecting membrane from the supernatant at 40,000 rpm for 1 hour at 4 °C. Membrane was resuspended with buffer B (20 mM Hepes pH 7.0, 300 mM NaCl, 10% (w/v) glycerol), and supplemented with final concentration is 1% LMNG-CHS (5:1). After solubilized at 4 °C for 2 hours, the solution was centrifuged at 20,000 rpm for 30 min, and the supernatant was loaded to Strep-Tactin^®^XT resin. The resin bound with protein was rinsed with buffer B containing 0.04% LMNG-CHS, and the target protein was eluted from the resin with buffer B containing 50 mM biotin. The crude protein was further purified by gel filtration with column superose 6. After gel filtration, the collections were used for preparing negative stains or cryo-EM grids. *Mt*EfpA mutants are purified according to the same protocol as the wide type. BRD8000.3 was added to the buffer for membrane solubilization and subsequent steps to acquire the *Mt*EfpA-BRD8000.3 complex. The monomeric *Mt*EfpA and *Ms*EfpA were purified with 1% DDM-CHS (5:1), instead of LMNG-CHS, and exchanged to amphipol PMAL-C8 before gel filtration for freezing cryo-EM grids as previously described^50^.

### Flow cytometry assay.

Cells with *Mt*EfpA constitutive expression, with empty expi293F as control, were collected, resuspended in PBS and aliquoted into three. One aliquot was fixed with 1% Paraformaldehyde (BioLegend). Another was incubated with APC anti-GFP antibody (BioLegend) followed by fixation. The third one was incubated with APC anti-GFP antibody, fixed, and permeabilized followed by incubating with APC anti-GFP antibody. All samples were made at least three parallel. Cell samples were assayed for FITC and APC with a CytoFLEX flow cytometer (Beckman). Data were analyzed with FlowJo Software (BD Biosciences).

### Negative stain analysis.

All negative-stain EM grids were made by applying 4 μL protein samples at 10 μg ml^−1^ to glow-discharged homemade grids. Negative-stain EM images were collected on a Tecnai T12 microscope (ThermoFisher) with pixel size of 1.68 Å. Automated particle picking from 3x binned images and 2D classification were performed with SAMUEL and SamViewer^51^.

### Cryo-EM specimen preparation and data collection.

All cryo-EM grids were prepared with Vitrobot Mark IV (Thermo Fisher Scientific) by application of 3 μL protein at 10 mg ml^−1^ to glow-discharged holey gold grid (Quantifoil, R1.2/1.3 μm, 300 mesh) and blotted 4 s with force −1 at 100% humidity and 4 °C before plunged into liquid ethane. Micrographs of *Mt*EfpAs were recorded on Talos Arctica or Titan Krios (ThermoFisher) operated at 200 kVe or 300 kVe with K3 direct electron detector (Gatan). SerialEM was used for automatic data collection with one shot per hole and nine holes per movement. The *Mt*EfpA dataset was collected with a pixel size of 0.87 Å, a total dose of 54 e Å^−2^, and 50 frames in total. The *Mt*EfpA-BRD8000.3 dataset was acquired in a super-resolution pixel size of 0.394 Å, 50 frames and total dose 49 e Å^−2^ with specimen stage tilt 20 degree. For monomeric *Ms*EfpA, two datasets were collected on Talos Arctica and Titan Krios with pixel size of 1.1 Å and 0.6632 Å, respectively. The defocus of all micrographs was set to range from −1.2 μm to −2.5 μm.

### Cryo-EM data processing.

3,465 movies of *Mt*EfpA were collected and were drift-corrected by MotionCorr2^52^. CTF information were estimated using CTFFind4^53^. 2,424,800 particles were picked and extracted from 3,031 selected micrographs with binning 4. Two rounds of 2D classification and deep2D classification were performed using SAMUEL^51^. After selection of good classes from deep2D, 450,607 particles are used for 3D classification and 3D refinement with C2 symmetry in Relion^54^. In the process of 3D classification and 3D refinement, as the resolution improved, we gradually replaced the binned particles with less binned or original dataset. Finally, a 3.1 Å resolution map of *Mt*EfpA in dimer was reconstructed by 49,783 particles with C2 symmetry after local refinement and postprocess.

For *Mt*EfpA complexed with BRD8000.3, the dataset was processed using cryoSPARC^55^. Briefly, after motion correction and CTF estimation, 2,014,853 particles were auto picked and extracted from 6,550 micrographs with binned 4-fold. Junk particles are removed by 2D classification, and 1,072,224 particles are re-extracted from micrographs with bin 2. After two rounds of ab-initio with setting maximum resolution were 6 Å and 4 Å, respectively, re-extraction selected particles without bin and supplement of sideview and top-view particles. Then the third round of ab-initio was performed with setting maximum resolution is 4 Å, following with non-uniform refinement^56^ and local refinement with applying C2 symmetry, a 3.3 Å map of mtEfpA-BRD8000.3 in outward state reconstructed by 52,099 particles are acquired finally.

For the two datasets of monomeric *Ms*EfpA, 6,546,828 particles and 3,142,841 particles were picked from two datasets with 3,280 and 3,554 micrographs, respectively, after motion correction and CTF estimation by cryoSPARC. After 2D classifications, 2,205,382 particles are selected and re-extracted with both pixel sizes are 1.1 Å before being combined. Then one round of ab-initio and two rounds of joint non-uniform refinement and non-alignment classification were performed with the combined particles. A 3.7 Å map of *Ms*EfpA was achieved in reconstructed by 52,150 particles after local refinement and post-process.

Gold standard Fourier Shell Correlation (FSC) = 0.143 ^57^ was applied to estimate the resolution of the final maps. Local resolutions of maps were calculated by RasMap^58^.

### Structural model building and determination.

To build *Mt*EfpA models, the AlphaFold-predicted model of *Mt*EfpA was cleaved into domains and docked into the 3.1 Å map of *Mt*EfpA in Chimera^59^. Then the model was adjusted with COOT^60^ and refined against the cryo-EM maps using real-space refinement in Phenix^61^. The structures and restraints of lipids and BRD8000.3 were generated by eBLOW^62^, then fitted into the map of *Mt*EfpA or *Mt*EfpA-BRD, respectively, following with real-space refinement and validated by MolProbity^63^. The *Ms*EfpA model was determined by molecular replacement with Phaser using *Mt*EfpA model as searching template before real-space refinement. All figures and movies were generated with Chimera or ChimeraX^64^.

### Shotgun lipidomic and small-molecule analysis.

Lipids and small molecule analytes were extracted using methyl tert-butyl ether (MBTE) and methanol^65^. Lipid-containing organic phase of each sample were transferred to new tubes and dried down under vacuum prior to mass spectrometry (MS) analysis. MS analysis of phospholipids were performed using an Orbitrap ID-X Tribrid mass spectrometer (Thermo Scientific), equipped with an automated Triversa Nanomate nanospray interface (Advion Bioscience) for lipid solution delivery with voltage of 1.4 kV over 6 minutes. All full MS1 scans of mass spectra were acquired in positive and negative modes using mass resolution of 500,000 (FWHM at m/z 200). Data independent acquisition of MS2 scans were performed in positive and negative modes at mass resolution of 30,000 (FWHM at m/z 500). Targeted MS1 scans were performed in both positive and negative modes with voltage of 1.4 kV for 6 minutes at resolution of 500,00 (FWHM at m/z 200) for *Mt*EpfA. Analyses of mass spectrometry data were performed using Xcalibur (Thermo Scientific) LipidXplorer 1.2.8 (CBG-MPI) software^66,67^.

### All-atom molecular dynamics (MD) simulations and docking.

The resolved structures of *Mt*EfpA and *Mt*EfpA-BRD were utilized as initial structures for MD simulations. The enveloping POPC lipid bilayer around *Mt*EfpAs was constructed using packmol-memgen program^68^ in AmberTools22^69^. The system was solvated in TIP3P water model with addition of 150 mM Na^+^ and Cl^−^ ions. The protein, ligands (CDL, PE, BRD) and POPC were parameterized using the AMBER ff14SB force field^70^, GAFF2 force field with AM1-BBC charge model ^71^ and Lipid21 force field^72^, respectively. After an initial energy minimization, the systems were initially heated to 100 K with fixed volume and further heated to 300 K, followed by pre-equilibration under isothermal-isobaric (NPT) conditions with decreasing positional restraints. Subsequently, a 100-ns NPT simulation without any restraints was performed, followed by a 500-ns production run. The temperature was maintained at 300 K using Langevin dynamics with a collision frequency of 2.0 ps^−1^, and the pressure was regulated using Monte Carlo barostat. The van der Waals and electrostatic interactions in real space were truncated at 10 Å, and the long-range electrostatic interaction was calculated using the particle mesh Ewald method. SHAKE was employed, and a time step of 2 fs was used in the above simulations, except for the final production simulation, in which hydrogen mass repartitioning^73^ was employed to enable a larger time step of 4 fs. All MD simulations were performed on GPUs using the pmemd.cuda module in the AMBER22. Four independent replicates of the simulations were performed using the same coordinates but different velocities.

BRD9327 was docked into *Mt*EfpA at the lipid binding site B using Smina^74^. The docking was carried out with the option exhaustiveness set to 96, and the top five poses returned by Smina were saved.

## Extended Data

**Extended Data Fig. 10 |:**
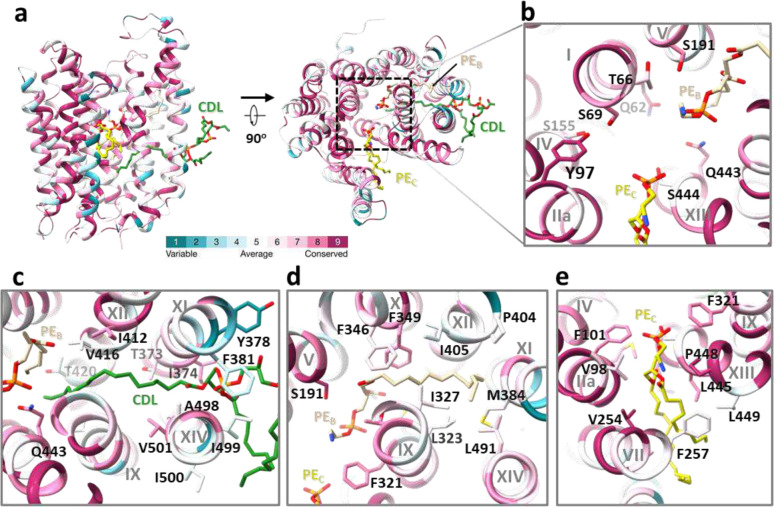
Conservation analysis of EfpA homologous in *Mycobacterium.* at the hydrophilic central pocket and three lipid-binding sites. **a**, Overall structure of *Mt*EfpA-lipids colored by conservation of 61 EfpA homologous from different species of *Mycobacterium*. **b-e**, Zoomed-in view of residue conservation at the hydrophilic central pocket (**b**) and three lipids binding sites (**c**-**e**).

**Extended Data Fig. 11 |:**
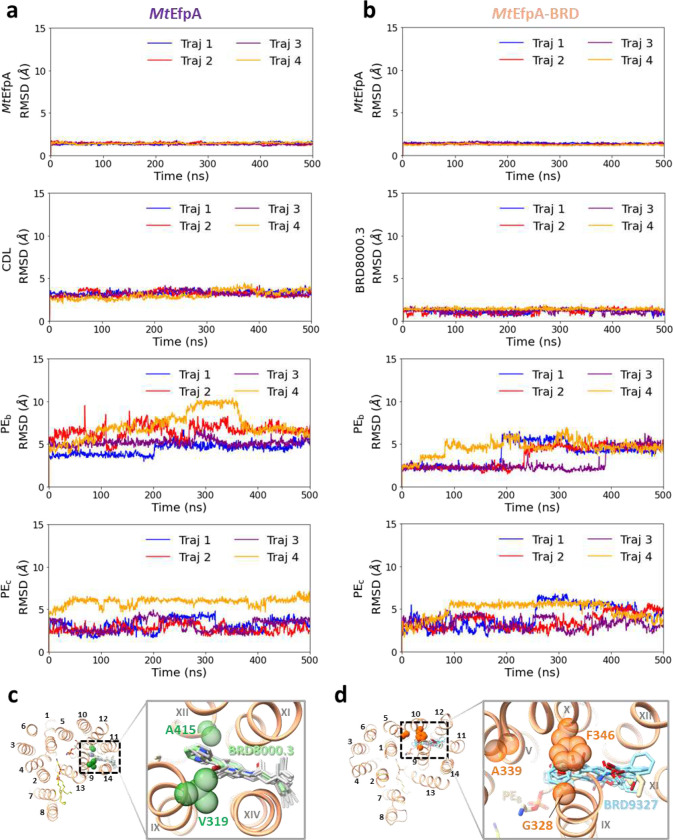
Molecular dynamics simulation analysis of ligands in *Mt*EfpA and Docking of BRD-9327. **a**, Molecular dynamics simulation analysis for *Mt*EfpA and RMSD of three lipids. **b**, Molecular dynamics simulation analysis for *Mt*EfpA-BRD8000.3 and RMSD of BRD-8000.3 and two lipids. **c**, The configuration of BRD-8000.3 in one of the trajectories extracted from every 50 ns of simulation (Gray) superimposed with the solved structure of *Mt*EfpA-BRD and the location of resistant mutations to BRD-8000.3 (Green). **d**, The location of resistant mutations to BRD-9327 (orange) on *Mt*EfpA and the top 5 docking results of BRD-9327 at lipid-binding site B.

**Extended Data Table 1|:** Data collection and refinement statistics

	*Mt*EfpA (EMD-37641) (PDB 8WM5)	*Mt*EfpA-BRD (EMD-42204) (PDB 8UFD)	*Ms*EfpA (EMD-42205) (PDB 8UFE)	

**Data collection and processing**				
Magnification	45,000	29,000	36,000	130,000
Voltage (kV)	200	300	200	300
Electron exposure (e−/Å^2^)	54	49	44	70
Defocus range (μm)	1.2–2.5	1.2–2.5	1.2–2.5	1.2–2.2
Pixel size (Å)	0.87	0.788	1.1	0.6632
Final Micrographs processed (no.)	3,031	6,550	3,280	3,554
Initial particle images (no.)	2,424,800	2,014,853	6,546,828	3,142,841
Final particle images (no.)	49,783	52,099	52,150	
Symmetry imposed	C2	C2	C1	
Map resolution (Å)	3.12	3.26	3.68	
FSC threshold	0.143	0.143	0.143	
**Refinement**				
Model resolution (Å)	3.2	3.5	4.1	
FSC threshold	0.5	0.5	0.5	
Model composition				
Non-hydrogen atoms	7179	7164	3449	
Protein residues	950	950	470	
Ligands	5	6	1	
*B* factors (Å^2^)				
Protein	55.14	101.10	114.57	
Ligand	55.79	102.03	105.33	
R.m.s. deviations				
Bond lengths (Å)	0.009	0.030	0.003	
Bond angles (°)	0.924	1.462	0.640	
Validation				
MolProbity score	1.88	1.90	2.19	
Clashscore	12.86	18.45	12.83	
Poor rotamers (%)	0.00			
Ramachandran plot				
Favored (%)	96.19	97.25	94.44	
Allowed (%)	3.81	2.75	5.34	
Disallowed (%)	0.00	0.00	0.21	

## Data Availability

The cryo-EM density maps have been deposited into the Electron Microscopy Data Bank under accession numbers EMD-37641 (*Mt*EfpA with PE and cardiolipin), EMD-42204 (*Mt*EfpA with PE and BRD8000.3) and EMD-42205 (*Ms*EfpA with PE). The atomic coordinates are deposited in the Protein Data Bank under accession codes PDB 8WM5 (*Mt*EfpA with PE and cardiolipin), 8UFD (*Mt*EfpA with PE and BRD8000.3) and 8UFE (*Ms*EfpA with PE). Source data for Fig. S1E is provided in the Source Data file.
